# Event and Comment

**Published:** 1914-05-15

**Authors:** 


// DENTAL REGISTER.

Vol. LXVIII. May 15, 1914. No. 5

### EVENT AND COMMENT.

ILLINOIS DENTAL SOCIETY. What will be regarded as one of the most conspicuous dental meetings ever held in this age or country was held in Chicago March 23rd to 26th, when the Illinois State Society celebrated its "Golden Anniversary Meeting." In the first place it breaks the record for attendance, more than four thousand dentists were registered on the evening of the second days session. This is fifteen hundred more than registered in the Chicago Dental Society Meeting several years ago, which up to this time held the attendance record. It was also unique as to the programme. There were comparatively few papers, but clinics, almost without number. The essays were presented in the evenings, and the daylight was fully occupied with various clinics. The first day was non-resident clinic day and clinicians were present from all parts of the United States and Canada, and the clinics were of an extraordinary high order, considering the large number presented. These were mostly table clinics, and the large assembly room of the La Salle Hotel was filled with clinicians and spectators for the entire day. It was practically impossible for any one person to see all these clinics, but we were surprised that we had a chance to see so many valuable clinics as we did. On the second day the Illinois dentists gave fifteen clinics by a new method which worked admirably. The spectators were divided into sections and assigned to a room, where a number of tables each accommodating ten to fifteen persons were placed, and as each of the subjects was presented, one person, who was the leader of as many other clinicians as there were tables in the room, gave a lecture, a paper or lantern exhibit, description of the clinic to be demonstrated by the men who presided at the several tables. The demonstrator by the use of models and other illustrations made clear to his section the paper or lecture as the case required. In this way every spectator had a chance to hear a well prepared description of the clinic and also to see it demonstrated by one of the demonstrators. It was interesting and should have been educational. On the third day the Chicago dentists were at home in their offices. Cards were issued to limited numbers who were privileged to call at the offices indicated at stated hours, where they were entertained by the dentists. The arrangements for handling in this way this large crowd was well planned and successfully carried out. It was a characteristic Chicago meeting, big and successful from all view points.

A pamphlet of 87 pages descriptive of the various clinics given by members of the Illinois State Society was a great help to enable every one to follow the clinics and will be good reference material. A special Committee prepared with great care another book of 300 pages containing a classified index of all the papers, discussions and clinics, with a biographic index of the officers and all other transactions of the society for the past fifty years. This will be of great value for future reference as much of Illinois history is of general importance. In fact this state has contributed very largely to dental progress in all lines during the past fifty years. We doubt if any other state could make so imposing a showing or put up such a programme as this latest achievement. We doubt however, if so large a meeting is of such general benefit in the way of imparting real instructions as the smaller meetings where more time and closer contacts are possible; but there is always generated in these large gatherings a certain amount of enthusiasm which is necessary for the cultivation of a spirit of emulation and progress. Let's have the big meetings to bring out the big guns, but don't neglect the small open courts where the modest fellows feel free to dip in and ask questions even though they hesitate to exhibit their attainments. We recently attended a local society in one of our small cities where about 30 dentists who knew each other well, held a three hour session of the greatest interests and value to every one present. It is in these smaller meetings that the future greats are developed, and if we are wise we shall give every possible encouragement to them in the way of personal and professional support. Our State Societies should start a propaganda of development of the local dental clubs and societies. This is one of the reasons for the great showing just made in Illinois.

MICHIGAN STATE DENTAL SOCIETY. The 58th annual meeting of this society was held in Detroit at the Hotel Tuller, April 9th to 11th. The meeting was unusually well attended. The programme was excellent, including some excellent papers and a good variety of high grade clinics. Ten papers were read and about 60 clinics were given. Eight of the clinics were given as progressive clinics. In these clinics the audience was divided into equal groups and were seated in a room where they remained while the clinicians moved from room to room until they had presented their clinics to each of the eight groups. This is a better plan than to have the audience follow up the clinicians, as there is less confusion and the audience is kept segregated until all have been served. The larger number of clinics however, were given in one large room and everyone was privileged to pick out his favorites. There are some serious objections to this old plan, but it is so strongly entrenched and is so natural a process, that it will take a good system of giving clinics to supplant it. We believe the clinician has much to do with the manner of presenting his subject. Some will do better with one method and others with another. We will get a better method some day with all the experiments we are now trying. One evening of the session was given over to a public meeting in the interest of public oral hygiene. Distinguished speakers gave interesting addressing showing that the general public is awakening to the value of good teeth for good health. Governor Ferris spoke on "The Duty of the State towards Oral Hygiene;" Dr. Vaughan, President of the American Medical Association, on "The Relation of Mouth Diseases to Public Health;" C. E. Chadsey, Sup't. Detroit Schools, on "The Attitude of Education towards Oral Hygiene in the Schools;" Dr. Price, Detroit Health Officer, on "The Status of Mouth Hygiene in Preventive Medicine;" Dr. C. H. Oakman, President Detroit Board of Health, on "What Detroit is doing in Mouth Hygiene;" Dr. W. A. White, State lecturer of New York State, on "What New York is doing for Mouth Hygiene in the Schools." There was a large audience of interested people, and we doubt not that much good will come from such a presentation.

There was no entertainment of a social character by the profession this time and we don't believe any one missed it, as every minute of the time was fully occupied with valuable proceedings to everyone. The programme was so varied that there was something for everyone.

The society decided unanimously to affiliate with the National Society with its entire membership of about 700.

A FOUR YEAR COURSE IN DENTISTRY. At the recent annual session of the Dental Faculties Association of American Univerities the seven schools constituting the membership of this organization decided unanimously to recommend to their several governing bodies the adoption of a four year curiculum as soon as it can be brought about. It is so obviously required for the adequate presentation of the ever increasing dental curiculum that there can be little question as to the propriety of the advancement. The only serious objection that has been raised heretofore was that the expense in time and money would materially reduce the number of students who would enter the profession, and just at this time there is an unusual demand for educated dentists. The position taken by these schools however, is that there is a more insistent demand for better educated dentists today than ever before and that it is the duty of the Universities to supply this demand first and demonstrate the fact that there is a sufficient number of thoughtful men to fully justify this increase in the opportunity for better preparation. Undoubtedly this will decrease the attendance in the schools adopting the four year course and increase the attendance in those requiring less time, so that there will probably be no net loss of graduate dentists.

There is some difference of opinion as to what should be included in the new curiculum, but it is the desire of everyone that some studies be added that will produce better thinkers as well as better doers. At present our scientific subjects are being crowded out by the technical, and unless we can controvert this tendency we shall have a one sided curiculum that will degenerate towards the craft to the detriment of the scientific. If our schools do not lay strongly the foundations for the future building of sound technical skill we can not hope for real advancement as a specialty of the healing art. The art of medicine is today more scientific than ever before, and the tendency in all medical training is toward the scientific in the medical colleges. This is a wise procedure, because scientific facts are more easily impressed on the younger mind, and when presented in logical sequence their cultural value can never be lost, and it is safe to predict a more satisfactory development of the clinical art when the mind has been properly prepared for discriminating selection. The dental as well as the medical practitioners will have all his career in which to develop the art side of his profession and it is safe to presume that he will develop a truer technique if he starts and builds from a scientific basis. We need therefore better scientific instruction for dentistry.

